# Protective Effect of Fermented Soybean Dried Extracts against TPA-Induced Oxidative Stress in Hairless Mice Skin

**DOI:** 10.1155/2013/340626

**Published:** 2013-08-29

**Authors:** Sandra R. Georgetti, Rúbia Casagrande, Fabiana T. M. C. Vicentini, Marcela M. Baracat, Waldiceu A. Verri, Maria J. V. Fonseca

**Affiliations:** ^1^Department of Pharmaceutical Science, Health Sciences Center, Londrina State University (UEL), Avenue Robert Koch 60, 86038350 Londrina, PR, Brazil; ^2^Department of Pharmaceutical Science, Faculty of Pharmaceutical Sciences of Ribeirao Preto (USP), Avenue Do Café s/n, 14040903 Ribeirao Preto, SP, Brazil; ^3^Department of Pathology, Biological Sciences Center, Londrina State University (UEL), Rodovia Celso Garcia Cid KM380 PR445, Campus Universitário, Caixa Postal 6001, 86051970 Londrina, PR, Brazil

## Abstract

This study evaluated the chemical properties (polyphenol and genistein contents) of soybean extracts obtained by biotransformation and dried by spray dryer at different conditions and their *in vivo* ability to inhibit 12-*O*-tetradecanoylphorbol-13-acetate- (TPA-) induced biochemical alterations in the skin of hairless mice. By comparing the obtained data with that of the well-known active soybean extract Isoflavin beta, we evaluated the influence of the fermentation and drying process in the extracts efficacy. The results demonstrated that inlet gas temperature and adjuvant concentration for the extract drying process have significantly affected the total polyphenol contents and, to a minor degree, the genistein contents. However, the effect of topical stimulus with TPA, an oxidative stress inducer, which caused significant depletion of reduced glutathione (GSH) and catalase, with increased levels of H_2_O_2_ and lipid peroxidation (MDA) in the skin of hairless mice, was significantly prevented by the soybean extracts treatment. These results indicate that the spray drying processing resulted in a product capable of limiting the oxidative stress with possible therapeutic applicability as an antioxidant in pharmaceutical forms.

## 1. Introduction

Skin, a biological environmental interface having a barrier function, is a potential target organ for oxidative stress by external offenders, such as UV irradiation, ozone, ionizing radiation, and various toxic chemicals [1].

The generation of reactive oxygen species (ROS) and their subsequent accumulation induces oxidative stress at the cellular level [2]. Although almost all organisms possess antioxidant defense and repair systems that have evolved to protect them against oxidative damage, these systems are insufficient to prevent the damage entirely. However, exogenous antioxidants, usually found in foods, can delay or inhibit the initiation or propagation of oxidative chain reactions [3]. Natural antioxidants in plants are related to three major groups: carotenoids, vitamins, and phenolics [4]. Phenolic compounds are plant-derived antioxidants that possess metal-chelating capabilities and radical-scavenging properties [5].

Soybean and soybean products, containing various compounds such as isoflavones, phytic acid, anthocyanin pigments, saponins, and unsaturated fatty acids, have also been postulated to protect against oxidative stress, due to their antioxidant ability [6].

The soybean isoflavones include genistein, daidzein, and biochanin A [7]. In many cases, the combined effect of soybean isoflavones might be better than the effect of a single isoflavone compound [8]. In addition, the aglycone forms of the isoflavones have higher biological activities [9], implying that isoflavone aglycone-rich products may be more effective than the ones rich in glycosides in preventing chronic diseases, such as coronary heart disease or cancer [10].

Brazil is one of the world's largest soybean exporters (Glycine max (Merrill) L.); however, soybean application as raw material for preparation of extracts for pharmaceutical use has been little employed by Brazilian industry and consequently the country is a major importer of soybean extract. On the other hand, in recent years, the industrial production of plant extracts is growing due to the worldwide phytopharmaceutical market trends and the increasing attention from the academic community and pharmaceutical companies [11].

Thus, the present study aimed to assess the *in vivo *protective effect of fermented soybean dried extracts and compare them with the well-known active soybean extract Isoflavin beta to evaluate the influence of fermentation and mainly the drying process in the extracts efficacy. For that, three different extracts were investigated as follows the soybean hydroalcoholic extract (HE) obtained by fermentation process with the fungus *Aspergillus awamori *and the spray-dried extracts (SE1 and SE2) obtained from HE employing different drying conditions. 

All the obtained extracts were firstly evaluated considering the chemical properties (polyphenol and genistein contents) and then their *in vivo* efficacy against the 12-*O*-tetradecanoylphorbol-13-acetate (TPA)-induced oxidative stress in the skin in view of the biochemical alterations: hydrogen peroxide (H_2_O_2_) formation, depletion of reduced glutathione (GSH) and catalase, and increase in (MDA) levels.

## 2. Materials and Methods

### 2.1. Chemicals and Microorganisms

Genistein, 5′-dithio-bis (2-nitrobenzoic acid) (DTNB), thiobarbituric acid (TBA), horseradish peroxidase (HRP), phenol red, and 12-*O*-tetradecanoylphorbol-13-acetate (TPA; CAS Number: 16561-29-8) were purchased from Sigma Chemical Co. (St Louis, MO, USA). Hydrogen peroxide 30% was purchased from Calbiochem (California, USA). Methanol, acetonitrile, acetic acid, gallic acid, and Folin-Ciocalteu reagent were from Merck (Darmstadt, Germany). Commercial soybean extract (Isoflavin beta) was obtained from Galena (Campinas, SP, Brazil) with at least 40% of isoflavones. All other chemicals were of reagent grade. The fungus used in this study was *Aspergillus awamori* (ATCC 22342).

### 2.2. Soybean Defatted Flour

Soybean seeds were of the Brazilian Doko variety, raised in the state of Parana (Brazil). Seeds dried at 37°C in a stove with air circulation were ground in a knife mill to fine particles (0.3 mm mean diameter). The soybean flour (1 part) was extracted with isopropanol (2 parts) by agitation for 2 h at room temperature. Defatted soybean flour was obtained by centrifuging the suspension at 3000 ×g for 30 min and drying the separated residue in a water bath.

### 2.3. Fermentation of Defatted Soybean Flour and Isoflavones Extraction

Starter cultures of microorganisms were previously activated by transference to potato dextrose agar (PDA, Oxoid, Basingstoke, UK) slants and incubation at 30°C for 5 days. Defatted soybean flour (10 g) was dispersed in 250 mL Erlenmeyer flasks with 10 mL of distilled water and autoclaved at 121°C for 15 min. Solid state fermentation was performed by evenly spraying 1.0 mL of spore suspension of the test organisms (10^7^ spores/mL) onto the autoclaved soybean substrate. After mixing, the inoculated soybean substrate was incubated for 48 h at 30°C [12].

Powdered samples (10 g) of unfermented and fermented soybean flour were extracted with an 80% aqueous methanol solution (1 : 10; w/v) under agitation for 2 h at room temperature. The homogenates were filtered through filter paper under vacuum, and the soybean hydroalcoholic extract (HE) was obtained.

### 2.4. Spray Drying

The drying was done in a spray dryer model SD-05 (Labplant, UK), with a concurrent flow regime. The drying chamber had a diameter of 215 mm and a height of 500 mm. The main components of the system were the feed system for HE, which consisted of a peristaltic pump, a two-fluid atomizer (inlet orifice diameter of 0.5 mm), and an air compressor; a feed system for the drying gas, which consisted of a blower and an air filter; a temperature control system for the drying gas; and a product collection system (cyclone). The gas humidity was measured at the inlet and at the exhaust of the spray dryer (SD) by means of a Traceables thermohygrometer. The inlet drying gas temperature was either 115 or 150°C, and the feed flow rate was kept constant at 4 g/min. The flow rate of the drying air was fixed at 0.0227 kg/s. The atomizing air feed flow rate was fixed at 15 L/min at a pressure of 1 bar [13]. The carrier agent used was colloidal silicon dioxide (tixosil 333) at concentrations of 45% and 70% for samples at 115°C (SE1) and 150°C (SE2), respectively.

### 2.5. Polyphenol Content

The HE was directly diluted 200 times with 50% ethanol. SE1 and SE2 (0.1 g) were stirred in 10 mL of 80% ethanol for 15 min. The ethanol suspensions were centrifuged at 1660 ×g for 10 min, and the supernatant fraction was collected into a 25 mL volumetric flask. The precipitate was again extracted with 5 mL of 80% ethanol. Finally, the supernatant fractions were combined and the volume was adjusted to 25 mL with deionized water. 

The total polyphenol content was determined by the Folin-Ciocalteu colorimetric method [14]. Approximately 0.5 mL of the soybean extracts solutions was mixed with 0.5 mL of the Folin-Ciocalteau reagent and 0.5 mL of 10% Na_2_CO_3_, and the absorbance was measured at 760 nm after 1 h incubation at room temperature. The total polyphenol contents were expressed as mg/g (gallic acid equivalents).

### 2.6. HPLC Determination of the Genistein Content

The genistein content in HE, SE1, and SE2 was determined by reverse-phase HPLC analysis. The HE was diluted 5 times in dimethylsulfoxide (DMSO) and then another 5 times in the mobile phase. Approximately 0.1 g of SE1 and SE2 was dissolved in 5 mL of DMSO and then diluted 5 times with the mobile phase. Aliquots of 20 *μ*L were injected into the HPLC system. Genistein separation was performed by employing a SuperPac Sephasil C18 (5 mm) column (250 × 4 mm), which was attached to a precolumn. The mobile phase consisted of 0.1% acetic acid in acetonitrile (35 : 65 v/v), which was used at a flow rate of 1 mL/min. Eluted isoflavonoid was detected by its absorbance at 250 nm. Quantitative data for genistein was obtained by comparing samples to a standard [15].

### 2.7. *In  Vivo* Evaluation of the Soybean Extracts Efficacy against Damage Induced by TPA

#### 2.7.1. Animals and Experimental Protocol

Sex-matched hairless mice (HRS/J), weighing 20–30 g, were housed in a temperature-controlled room, with access to water and food *ad libitum* until use. All experiments were conducted in accordance with National Institutes of Health guidelines for the welfare of experimental animals and with the approval of the Ethics Committee of the Faculty of Pharmaceutical Science of Ribeirao Preto (University of Sao Paulo).

To study the effect of soybean extracts on TPA-mediated cutaneous oxidative stress, 18 mice were randomly allocated to 6 groups of three mice each. Each of the groups were exposed to a different treatment as follows (1) topical application of 0.2 mL acetone (TPA vehicle control) for three days; (2) only TPA; (3) topical application of 200 *μ*g HE (based solid total)/0.2 mL acetone/animal for three days; (4) topical application of 200 *μ*g SE1/0.2 mL acetone/animal for three days; (5) topical application of 200 *μ*g SE2/0.2 mL acetone/animal for three days; and (6) topical application of 200 *μ*g Isoflavin beta/0.2 mL acetone/animal for three days. One hour after their last treatment with the soybean extracts on the third day, the animals from groups 2–6 received the topical application of TPA (20 nmol/0.2 mL acetone/animal). Mice were sacrificed 2 h after TPA stimulus [8], and the skin tissues were excised and washed with ice-cold 0.9% NaCl. The tissues were homogenized in cold 0.1 M phosphate buffer (pH 7.4) using a Polytron homogenizer (PT3100). After that, homogenates were centrifuged at 12100 ×g for 20 min [13], and the resulting supernatant was used for further analysis. The results are representative of three experiments with 3 mice per group in each experiment.

#### 2.7.2. Estimation of Hydrogen Peroxide

Hydrogen peroxide levels in skin samples were estimated by the method described by Sharma and Sultana [8] with modification by Georgetti et al. [13]. Briefly, a 0.5 mL solution of 0.1 mg/mL phenol-red in phosphate buffer (pH 7.4; 0.1 M) and 50 *μ*g/mL horseradish peroxidase in the same buffer were mixed with 0.5 mL of different skin supernatant fractions and incubated at 37°C for 10 min. Subsequently, 1 mL of 1 M NaOH was added, and the absorbance was measured at 610 nm. Hydrogen peroxide was calculated as nmol H_2_O_2_/g tissue using a molar extinction coefficient of 43600 M^−1 ^cm^−1^.

#### 2.7.3. Estimation of GSH

The concentration of reduced glutathione (GSH) in skin samples was determined as previously described by Saleem et al. [16]. Briefly, 1 mL of the different skin supernatant fractions was mixed with 1 mL of 4% sulphosalicylic acid. The samples were incubated at 4°C for at least 1 h and then centrifuged at 1200 ×g for 15 min at 4°C. The reaction mixture contained 0.4 mL of the filtered sample, 2.2 mL of phosphate buffer (pH 7.4; 0.1 M), and 0.4 mL of 4 mg/mL of 5′-dithio-bis (2-nitrobenzoic acid) (DTNB) in a total volume of 3 mL. The yellow color developed was read immediately at 412 nm. The GSH concentration was calculated as nmol GSH/g tissue using a molar extinction coefficient of 13700 M^−1 ^cm^−1^.

#### 2.7.4. Catalase Activity

Catalase activity was measured using the method described by Claiborne [17]. The reaction mixture consisted of 2 mL of phosphate buffer (pH 7.4; 0.1 M), 0.95 mL of 0.019 M hydrogen peroxide, and 0.05 mL of the different skin supernatant fractions in a final volume of 3 mL. Changes in absorbance were recorded at 240 nm. Catalase activity was calculated as nmol H_2_O_2_ consumed/min/mg protein using a molar extinction coefficient of 43600 M^−1 ^cm^−1^.

#### 2.7.5. Estimation of Lipid Peroxidation

The assay for lipid peroxidation was performed according to a method described by Iqbal et al. [18] and Georgetti et al. [13]. The reaction mixture consisted of 0.58 mL of phosphate buffer (pH 7.4; 0.1 M), 0.2 mL of the different skin supernatant fractions, 0.2 mL of 100 mM ascorbic acid, and 0.02 mL of 100 mM ferric chloride in a total volume of 1 mL. The mixture was incubated at 37°C for 1 h. The reaction was stopped by the addition of 1 mL of 10% trichloroacetic acid. All tubes were placed in a boiling water bath for 20 min, followed by an ice bath and centrifugation at 2500 ×g for 10 min. The amount of MDA formed in each sample was assessed by measuring the absorbance at 535 nm. The results were expressed as nmol MDA formed/h/g tissue at 37°C using a molar extinction coefficient of 1.56 × 10^5^ M^−1 ^cm^−1^ [13, 16].

### 2.8. Statistical Analysis

Data were statistically analyzed by one-way ANOVA, followed by Bonferroni's multiple comparisons *t*-test for evaluation of the effect of different soybean extracts against TPA-induced oxidative damage in mice skin. Results were presented as the mean ± S.E.M. (standard error mean) and considered significantly different when a *P* < 0.05 was obtained. Statistical analyses were performed using GraphPad Prism 4.0 software (GraphPad Software, Inc. San Diego, California, USA).

## 3. Results and Discussion

Soybeans contain various important phenolic compounds, including free phenolic acid, phenolic acid esters, the isoflavones genistein and daidzein (and their glycosides), and coumestrol [19]. In soy foods, isoflavones are present mainly as glucosides. In general, the aglycone forms of isoflavone present higher biological activity than the glycone forms [9]. This implies that products rich in aglycone isoflavones may be more effective than those rich in isoflavone glycosides at preventing chronic diseases [10]. In agreement with this approach, there is evidence that fermentation increases the amounts of aglycone forms of isoflavones and their antioxidant activities [9, 12]. Therefore, in our previous study [12], the fermentation process was employed to obtain more active soybean extracts, and based on this the HE, which presented elevated antioxidant activities due to increased amounts of aglycone isoflavones compared to the unfermented extract, was selected. This extract (HE) was the starting material for obtaining, in the present study, two different dried extracts: SE1 (dried at 115°C with 45% of adjuvant) and SE2 (dried at 150°C with 70% of adjuvant). 

The aim of drying an extract is to increase its chemical and microbiological stability. Furthermore, dried extracts are known to be dosed more accurately and are more easily stored and transported than the corresponding liquid forms. Consequently, the dried forms can be used as either an intermediate or final product [20]. The dried extracts are considered technologically feasible for large scale production due to their greater stability and ease in standardization of their active principles [20, 21]. 

The commercial extract Isoflavin beta, with known activity, was used as a reference standard.

First, the total polyphenols and genistein contents in the hydroalcoholic extract and fermented soybean spray dried extracts were evaluated (Table 1). Significant reduction in the total polyphenols content (60%) was detected in SE1 and SE2 compared to HE. However, there was no statistically significant difference between SE1 and SE2. These results suggest that the phenolic compounds present in the HE might be degraded by the spray drying process independent of the employed drying parameters. Other studies also detected the degradation of polyphenols after spray drying and suggested that it may have been caused by oxidative condensation phenomena and decomposition of thermolabile compounds induced by in-process factors such as heating [11, 22, 23].

In addition to the polyphenols content, the aglycone isoflavone genistein content was also used as a quality indicator of the extracts since it is the major isoflavone in soybeans and has exhibited a variety of anticancer properties [24]. Previous studies have demonstrated that genistein inhibits 12-*O*-tetradecanoylphorbol-13-acetate induced H_2_O_2_ formation, inflammatory responses and protooncogene expression, and skin tumorigenesis [25]. In addition, it was also demonstrated that genistein blocked oxidative DNA damage induced by UVC and benzo(a)pyrene plus UVA [26] and inhibited UVB-induced protooncogene expression and skin tumorigenesis in hairless mice [25, 27]. 

A statistically significant difference between the genistein contents of HE and SE1 was detected, but not between HE and SE2. Furthermore, SE2 presented higher genistein content than SE1 (Table 1). These results might suggest that the higher concentration of adjuvant in SE2 (70%) compared to SE1 (45%) might be protecting the isoflavone genistein from the degradation process during spray drying. 

The total polyphenol and genistein contents in the commercial soybean extract Isoflavin beta were 75 mg/g and 87 mg/g, respectively, as previously determined by our group [28]. These values are much greater than those found for all the extracts evaluated in the present study. 

According to Nicoli et al. [29], the changes that occurred during the processing are expected to affect the content, activity and bioavailability of the bioactive compounds. In addition, compounds capable of providing a synergistic effect for antioxidant activity could be damaged or removed due to the drying, contributing for the reduction of the antioxidant effectiveness of the dried product [30–32]. Therefore, we addressed the possible effects of the spray drying process on the extracts effectiveness by comparing the *in vivo* activity of HE, SE1, SE2, and the commercial extract Isoflavin beta, on a model of TPA-induced oxidative stress. 

TPA is a potent tumor promoter and has been reported to act through the generation of ROS [33] and cause a concomitant decrease in the antioxidant status of the cells [34]. It is recognized as an inflammatory agent [2] and tumor promoter that has been shown to generate superoxide anion, hydrogen peroxide, and lipid hydroperoxides, leading to oxidative stress. Topical application of TPA on mouse skin resulted in discernible oxidative response as measured by increased hydrogen peroxide and MDA production and depletion of antioxidant defense like catalase [8] and GSH. 

In the present study, the protective effect of the fermented soybean dried extracts against the TPA-induced oxidative stress in the skin was evaluated considering the biochemical parameters: H_2_O_2_, reduced glutathione, catalase, and MDA levels.

TPA application results in significant increase in H_2_O_2_ production on skin, and a close relationship between the generation of ROS, including superoxide dismutase (O_2_^•−^), and tumor promotion has been observed [35]. Abnormal production of H_2_O_2_ is also known to initiate phosphorylation of mitogen-activated protein (MAP) kinase and activation of its downstream signals resulting in the expression of genes having direct relevance to the carcinogenesis process [36]. 

**Figure 1:**
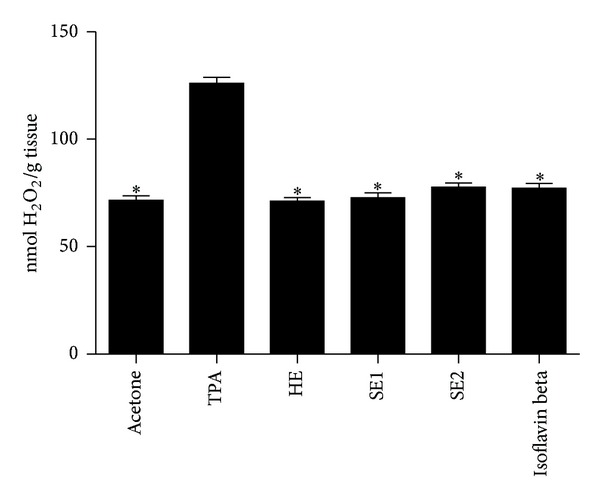
Role of pretreatment with different soybeans extracts on TPA-induced H_2_O_2_: hydroalcoholic extract (HE), spray-dried soybean extract at 115°C/45% (SE1), and spray-dried soybean extract at 150°C/70% (SE2). Bars represent means ± SD of three separate experiments with 3 animals per group per experiment.*Significant (*P* < 0.05) when compared with TPA treated control group.

The results demonstrated that the application of TPA increased the skin susceptibility to H_2_O_2_ generation by 76% when compared with the levels found in the control group (treated with vehicle). Nevertheless, the skin pretreatment with HE, SE1, SE2, and Isoflavin beta maintained the skin H_2_O_2_ content close to the control group (Figure 1). 

**Figure 2:**
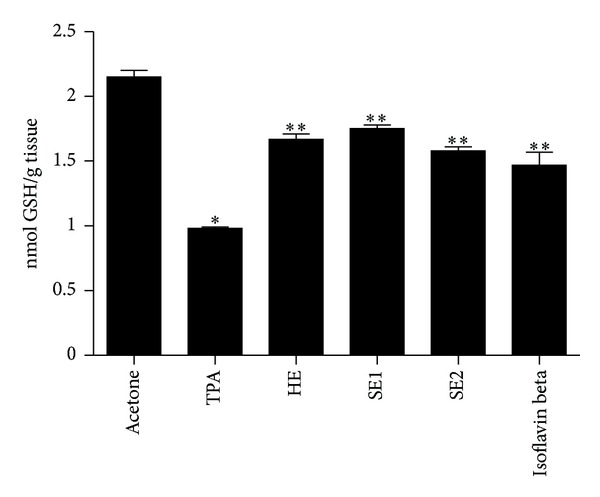
Role of pretreatment with different soybean extracts on TPA-induced GSH depletion: hydroalcoholic extract (HE), spray-dried soybean extract at 115°C/45% (SE1), and spray-dried soybean extract at 150°C/70% (SE2). Bars represent means ± SD of three separate experiments with 3 animals per group per experiment. *Significant (*P* < 0.05) when compared with acetone treated control group. **Significant (*P* < 0.05) when compared with acetone treated control group and TPA treated control group.

The GSH directly scavenges radicals by hydrogen transferring and acts as a cofactor for the enzyme GSH-peroxidase, which in turn scavenges peroxides finally regenerating vitamins E and C [37]. Treatment of animals with TPA alone resulted in a 54.4% depletion of cutaneous glutathione. Nevertheless, the pretreatment of animals with HE, SE1, SE2, and Isoflavin beta prior to the application of TPA inhibited the GSH depletion in the range of 42–65.8% (Figure 2). The recovery of the depleted GSH did not show significant statistical difference (*P* < 0.05) between the soybean extracts; however, the commercial extract Isoflavin beta showed the lowest recovery value of GSH levels. 

**Figure 3:**
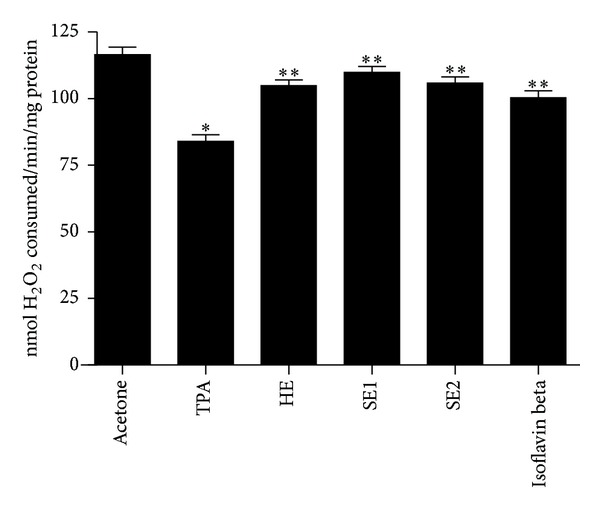
Role of pretreatment with different soybeans extracts on TPA-induced catalase depletion: hydroalcoholic extract (HE), spray-dried soybean extract at 115°C/45% (SE1), and spray-dried soybean extract at 150°C/70% (SE2). Bars represent means ± SD of three separate experiments with 3 animals per group per experiment. *Significant (*P* < 0.05) when compared with acetone treated control group. **Significant (*P* < 0.05) when compared with TPA treated control group.

In addition, the effects of pretreatment with soybean extracts on TPA-mediated depletion in the level of catalase are showed in Figure 3. Treatment with only TPA resulted in approximately 28% reduction of cutaneous catalase when compared with the vehicle-treated control group. The recovery of this antioxidant enzyme was 65.0, 77.0, 67.5, and 50.5% for HE, SE1, SE2, and Isoflavin beta, respectively.

By comparing the efficacy of the extracts against TPA-induced GSH and catalase depletion a similar response profile, can be observed, with all the proposed extracts being more active than the commercial available Isoflavin beta.

**Figure 4:**
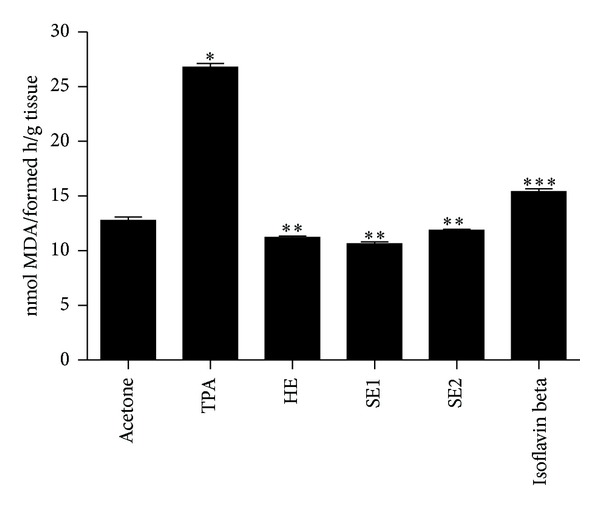
Effects of pretreatment with different soybeans extracts on TPA-mediated enhancement in the susceptibility of cutaneous skin for iron ascorbate-induced lipid peroxidation in mice: hydroalcoholic extract (HE), spray-dried soybean extract at 115°C/45% (SE1), and spray-dried soybean extract at 150°C/70% (SE2). Bars represent means ± SD of three separate experiments with 3 animals per group per experiment. *Significant (*P* < 0.05) when compared with acetone treated control group. **Significant (*P* < 0.05) when compared with acetone treated control group and TPA treated control group. ***Significant (*P* < 0.05) when compared with TPA treated control group and HE, SE1, and SE2 treated groups.

Finally, the effects of pretreatment with different soybean extracts on TPA-mediated susceptibility of cutaneous mice skin to iron-ascorbate-induced lipid peroxidation are showed in Figure 4. The levels of MDA were increased by approximately 2-fold in the mice skin after treatment with TPA alone. The pretreatment of skin with soybean extracts reduced the lipid peroxidation close to the value found in the acetone-treated control group, and again the least active was the Isoflavin beta. In this case a statistical significant difference between the MDA levels found for the Isoflavin beta-treated group, and the HE, SE1, and SE2-treated groups was detected. 

Boadi et al. [38] have demonstrated that genistein inhibited the oxidation of methyl linolenate via Fenton's pathway and might have chelated the metal ions or donated hydrogen atoms to neutralize the hydroxyl radicals. Additionally, Toda and Shirataki [39], by evaluating the inhibitory effects of four isoflavones (biochanin A, daidzein, formononetin, and genistein) on lipid peroxidation by reactive oxygen species, have demonstrated that the differences in antioxidant activities found are dependent on the relation between their chemical structures and the different reactive oxygen species formed. Therefore, the use of soybean extracts, which are composed of different isoflavonoids, is advantageous over an isolated compound, once the LPO inhibitory activity can be caused through antioxidant activity in different reactive oxygen species.

Interestingly, although the SE1 and SE2 presented lower concentration of total polyphenols than the HE, they showed similar *in vivo* efficacy in all the experimental models. According to Mrkìc et al. [40], the oxidation reactions may take place during drying and polyphenols with an intermediate oxidation state can exhibit higher radical scavenging activity than nonoxidized polyphenols. High temperature drying could further cause the formation of the Maillard reaction products (MRPs) that have been shown to act as antioxidants in dried foodstuffs, individually or in synergism with the naturally occurring antioxidants. Thus, the loss of natural antioxidants in heated foods could be minimized or compensated by the formation of nonnutrient antioxidants such as MRPs, enhancing the overall antioxidant properties of the product [29].

Furthermore, the present study found that our extracts (HE, SE1, and SE2) were better than the commercial extract Isoflavin beta in inhibiting the TPA-induced oxidative stress in the skin of hairless mice, despite the latter having greater total polyphenol and genistein contents. Similarly our extracts also showed similar or higher efficacy in inhibiting different TPA-induced biochemical alterations than the commercial soybean extract Novasoy evaluated by Sharma and Sultana [8], despite its greater genistein content (300 *μ*g/g). 

These results indicate that the activity observed by our extracts may be related not only to the polyphenols and genistein contents, but also to a synergism that can occur between these and the other components that are present in the extracts. 

## 4. Conclusions

The present study showed that despite the fact that the soybean fermented and dried extracts resulted in less content of total polyphenol and genistein than the commercial available Isoflavin beta, they have demonstrated higher *in vivo* efficacy against the biochemical alterations induced by TPA application, particularly in inhibiting the lipid peroxidation. These results may indicate the presence of other components in the extracts, such as peptides produced during the fermentation, which can synergistically provide better biological activity.

Likewise, the results obtained demonstrate that although the employed drying process has significantly affected the total polyphenol contents and, to a minor degree, the genistein contents, the efficacy was maintained. Therefore, spray drying appears to be an adequate method for drying soybean extracts, and increasing their stability and possible commercial/industrial application while maintaining their *in vivo *activity to be used in pharmaceutical forms to limit the oxidative stress. 

## Figures and Tables

**Table 1:** Total polyphenol and genistein contents in HE, SE1, and SE2.

Samples	Total polyphenol (mg/g total solids)	Total genistein (*µ*g/g total solids)
HE	59.60 ± 0.085	136.57 ± 2.96
SE1	21.13 ± 1.13^a^	122.90 ± 2.76^ab^
SE2	22.29 ± 1.31^a^	138.02 ± 2.34

^a^Significant statistical difference compared to HE, ^b^significant statistical difference compared to dried extract SE2. HE results were calculated based on total solids.
